# Burnout Among Medical Staff 1 Year After the Beginning of the Major Public Health Emergency in Wuhan, China

**DOI:** 10.3389/fpsyg.2022.893389

**Published:** 2022-07-05

**Authors:** Wenning Fu, Yifang Liu, Keke Zhang, Pu Zhang, Jun Zhang, Fang Peng, Xue Bai, Jing Mao, Li Zou

**Affiliations:** ^1^School of Nursing, Tongji Medical College, Huazhong University of Science and Technology, Wuhan, China; ^2^Department of Neurology, Zhongnan Hospital of Wuhan University, Wuhan, China; ^3^Department of Cardiology, Taihe Hospital, Hubei University of Medicine, Shiyan, China; ^4^Department of Endocrinology, Taihe Hospital, Hubei University of Medicine, Shiyan, China; ^5^Reproductive Medical Center, Renmin Hospital of Wuhan University, Wuhan, China

**Keywords:** medical staff, burnout, mental resilience, major public health emergency, Wuhan

## Abstract

**Objectives:**

Wuhan is the city where coronavirus disease (COVID-19) was first reported and developed into a pandemic. However, the impact of the prolonged COVID-19 pandemic on medical staff burnout remains limited. We aimed to identify the prevalence and major determinants of burnout among medical staff 1 year after the beginning of the COVID-19 pandemic in Wuhan, China.

**Materials and Methods:**

A total of 1,602 medical staff from three hospitals in Wuhan, China, were included from November 1–28, 2021. Chi-square tests were conducted to compare the prevalence of burnout across groups based on sociodemographic and professional characteristics. A multivariate analysis was performed using a forward stepwise logistic regression model.

**Results:**

Approximately 37.39% of the medical staff experienced burnout 1 year after COVID-19 pandemic. Emotional exhaustion (EE) was the most common symptom of burnout, with 1,422 (88.76%) participants reporting a severe EE. Burnout was associated with insufficient social support and “neutral” or “dissatisfied” patient-physician relationships. Respondents who participated in the care of COVID-19 patients had a higher risk of burnout symptoms than those who did not participate. In particular, mental resilience was negatively associated with burnout among the medical staff.

**Conclusion:**

Nearly two-fifths of the participants had symptoms of burnout, with reduced personal accomplishment being the predominant symptom 1 year after COVID-19. Healthcare organizations should regularly measure and monitor burnout among the medical staff. In addition, creating positive work environments and improving the mental resilience of medical staff may be effective ways to reduce burnout.

## Introduction

Coronavirus disease 2019 (COVID-19) has been a major public health emergency, with a significant physical and mental impact on the population. Burnout is defined as the psychological syndrome of emotional exhaustion (EE), cynicism (CY), and reduced professional accomplishment (Maslach et al., 2001). It is not only one of the most common occupational phenomena but also a psychological disorder with serious consequences for individuals and society (Panagioti et al., 2017; Meynaar et al., 2021). Additionally, burnout is significantly related to anxiety, depression, sleep disturbances, substance abuse, turnover intention, and decreased job satisfaction (Shanafelt et al., 2012; Guo et al., 2018; Patel et al., 2018; Zhang et al., 2020). Medical staff experiencing burnout are more likely to provide suboptimal patient care and make more medical errors (Mealer et al., 2009; Panagioti et al., 2018).

The COVID-19 pandemic has created a perfect storm for the mental health of medical staff (Restauri and Sheridan, 2020). Burnout from chronic work stress intersects with acute traumatic stress from the COVID-19 pandemic, which may exacerbate psychological distress in the medical staff. Under the circumstances of increased work intensity, prolonged working hours, and widespread panic caused by the COVID-19 outbreak, medical staffs are at increased risk of developing burnout (Barello et al., 2020; Morgantini et al., 2020). Burnout among medical staff has been exacerbated and needs to be addressed.

A survey of nurses during the infectious SARS epidemic showed that the epidemic had a long-term effect on burnout among medical staff (Liu et al., 2012). To date, there has been no definite estimate of the COVID-19 pandemic duration. Understanding burnout among medical staff is necessary to improve the quality of care while ensuring an adequate healthcare workforce to overcome COVID-19. However, the long-term impact of COVID-19 on burnout among medical staff remains uncertain. In particular, similar studies on burnout among Chinese medical staff 1 year after the start of the COVID-19 pandemic are limited.

Therefore, the overall purpose of this study was to comprehensively assess the status of burnout among medical staff 1 year after the outbreak of the COVID-19 pandemic in terms of individual social factors, the impact of COVID-19, mental resilience, and the level of social support. More specifically, we aimed to address the following: (1) the distribution characteristics of burnout symptoms among medical staff 1 year after the COVID-19 pandemic in Wuhan, China, and (2) the analysis of potential factors influencing burnout among health staff. These findings provide a reference for the management of burnout among the medical staff.

## Materials and Methods

This study was approved by the Research Ethics Committee of Tongji Medical College, Huazhong University of Science and Technology, Wuhan, China. This multicenter study was conducted from June 1 to June 28, 2021, in Wuhan, China. The Strengthening the Reporting of Observational Studies in Epidemiology (STROBE) guidelines for cross-sectional studies were used in this study (Von Elm et al., 2007).

The sampling process in this study consisted of two stages. In the first stage, three hospitals in Wuhan were randomly selected using a simple random sampling principle. In the second stage, online questionnaires were sent to the medical staff of the three selected hospitals through the department of medical office and nursing department. A total of 1,602 medical staff members were included in the study, with a response rate of 97.09%. All participants completed a questionnaire on an online survey platform (“SurveyStar,” Changsha Ranxing Science and Technology, Shanghai, China). Prior to registration, all participants provided electronic informed consent. Two options (yes/no) are available on the informed consent page. Only participants who picked “yes” were included on the next page. Each verified account was eligible to answer once to prevent repeated questionnaires. Simultaneously, intelligent logic examinations were set up in the software to identify inoperative questionnaires. Two independent researchers checked the answers to all valid questionnaires, which were automatically entered into a data file.

### Measurements

A structured questionnaire was designed to identify factors that may be associated with burnout, including demographic information (sex, age, marital status, educational level, monthly income, occupation, and work department), professional characteristics (job title, night-shift work, patient-nurse-physician, whether directly participating in the care of patients with COVID-19), mental resilience, and social support.

Mental resilience was assessed using the 10-item Connor–Davidson Resilience Scale (CD-RISC-10) (Campbell-Sills and Stein, 2007). The scale consists of ten items reflecting an individual’s ability to tolerate experiences, such as change and pressure. Each item is scored on a 5-point Likert scale, ranging from “never” to “almost always,” coded with values from 0 to 4. Possible scores ranged from 0 to 40, with higher scores indicating greater resilience capacity. The CD-RISC-10 showed satisfactory reliability and validity in the Chinese sample with the Cronbach’s alpha of 0.92 (Cheng et al., 2020). The Cronbach’s alpha of the CD-RISC-10 in this study was 0.977.

Social support was measured using the multidimensional scale of perceived social support (MSPSS) (Zimet et al., 1988). The scale contains 12 items, with response options ranging from 1 (very strongly disagree) to 7 (very strongly agree), measuring the extent to which each item was experienced. The total scores ranged from 12 to 84, with higher scores representing higher levels of social support. An MSPSS score of 12–36 suggests “low-level social support”; 37–60, “medium-level social support”; and 61–84, “high-level social support.” The Chinese version of MSPSS has excellent reliability and validity with the Cronbach’s alpha of 0.89 (Chou, 2000), so the scale is widely used in China (Yu et al., 2020; Zhao et al., 2022). In the current study, the Cronbach’s alpha was 0.979.

Burnout was measured using the Chinese version of the Maslach burnout inventory-general survey (MBI-GS) (Li, 2003). The scale has 15 items with three dimensions, emotional exhaustion (EE, five items), cynicism (CY, four items), and reduced personal accomplishment (PA, six items). Each item was scored on a 7-point Likert scale ranging from “never” to “every day,” coded with values from 0 to 6. Items related to personal accomplishment were reverse scored (reduced PA). The three dimensions were scored separately. Higher scores indicate the presence of more symptoms. The cut-off points for the MBI-GS subscales were as follows: EE (low < 17; average, 17–25; high > 25), CY (low < 7; average, 7–11; high > 11), and reduced PA (low < 12; average, 12–16; high > 16) (Lyl, 2006). Based on the scores of >25 for EE, >11 for CY, and >16 for reduced PA, individuals were classified as “no burnout” (each of the three dimensions scored below the corresponding cut-off points), “mild burnout” (only one dimension scored above the corresponding cut-off points), “moderate burnout” (any two dimensions scored above the corresponding cut-off points), and “severe burnout” (all three dimensions scored above the corresponding cut-off points).

### Statistical Analysis

All analyses were performed using Statistical Analysis System (SAS) 9.4 for Windows (SAS Institute Inc., Cary, NC, United States). Participants’ sociodemographic and professional characteristics and the levels of burnout were described using frequency and percentage. We divided the participants into two groups for the remaining analyses: “no burnout” and “burnout” (mild burnout, moderate burnout, and severe burnout combined). Chi-square tests were conducted to compare the prevalence of burnout across groups based on sociodemographic and professional characteristics. A multivariate analysis was performed using a forward stepwise logistic regression model. All comparisons were two-tailed, and p-values less than 0.05 were considered statistically significant.

## Results

### Sociodemographic Characteristics of Participants

Table 1 shows the participants’ characteristics and the prevalence of burnout 1 year after the COVID-19 outbreak. A total of 1,602 medical staff members participated in the survey. Of these, 86.64% were women, 72.78% were married, and 86.58% reported college or higher education levels. Nearly 90% of the respondents were nurses, and 80.34% reported that they were satisfied with patient-physician relationships. Approximately half of the respondents directly participated in the care of patients with COVID-19.

**TABLE 1:** Participants’ characteristics and associations with job burnout in medical staff.

Variables	Total (*N* = 1,602)		Burnout (*N* = 599)		No burnout (*N* = 1,003)		*P*-value
	*n*	%	*n*	%	*n*	%	
**Gender**							
Male	214	13.36	91	15.19	123	12.26	
Female	1,388	86.64	508	84.81	880	87.74	0.0955
**Age**							
20–29	500	31.21	196	32.72	304	30.31	
30–39	805	50.25	315	52.59	490	48.85	0.0092
≥40	297	18.54	88	14.69	209	20.84	
**Marital status**							
Married	1,166	72.78	446	74.46	720	71.78	
Single	400	24.97	142	23.70	258	25.73	0.4280
Divorced/Widowed	36	2.25	11	1.84	25	2.49	
**Educational level**							
Without college education	215	13.42	87	14.52	128	12.76	
College and above	1,387	86.58	512	85.48	875	87.24	0.3167
**Monthly income (RMB)**							
<5,000	468	29.21	188	31.39	280	27.92	
≥5,000	1,134	70.79	411	68.61	723	72.08	0.1395
**Occupation**							
Doctor	139	8.68	57	9.52	82	8.18	
Nurse	1,400	87.39	514	85.81	886	88.33	0.3028
Others	63	3.93	28	4.67	35	3.49	
**Work department**							
Internal medicine	421	26.28	161	26.88	260	25.92	
Surgical department	442	27.59	156	26.04	286	28.52	0.5635
Others	739	46.13	282	47.08	457	45.56	
**Job title**							
Elementary or less	802	50.06	315	52.59	487	48.55	
Intermediate/Senior	800	49.94	284	47.41	516	51.45	0.1183
**Night-shift work**							
Yes	1,092	68.16	398	66.44	694	69.19	
No	510	31.84	201	33.56	309	30.81	0.2532
**Patient-physician relationship**							
Satisfied	1,287	80.34	428	71.45	859	85.64	
Neutral/Dissatisfied	315	19.66	171	28.55	144	14.36	<0.0001
**Whether you directly participated in the care of patients with COVID-19 or not**							
No	786	49.06	257	42.90	529	52.74	
Yes	816	50.94	342	57.10	474	47.26	0.0001

### Prevalence and Symptoms of Burnout Among the Medical Staff 1 Year After the COVID-19 Pandemic

The overall prevalence of burnout symptoms among the medical staff was 37.39% (599/1,602), with the prevalence of “mild,” “moderate,” and “severe” burnout at 33.77, 3.43, and 0.19%, respectively (Table 2).

**TABLE 2:** Prevalence of burnout at different levels among medical staff.

Categories	*n*	%
No burnout	1,003	62.61
Mild burnout	541	33.77
Moderate burnout	55	3.43
Sever burnout	3	0.19

Table 3 shows the three dimensions of burnout symptoms among the medical staff. The prevalence of low EE, CY, and reduced PA was 88.76, 83.21, and 57.93%, respectively, 1 year after the beginning of the COVID-19 pandemic. The average prevalence of EE, CY, and reduced PA was 8.99, 10.11, and 9.80%, respectively. The prevalence rates of high EE, CY, and reduced PA were 2.25, 6.68, and 32.27%, respectively (Table 3).

**TABLE 3:** Prevalence of three dimensions of burnout at different levels among medical staff.

	Low		Average		High	
	*n*	%	*n*	%	*n*	%
EE^a^	1,422	88.76	144	8.99	36	2.25
CY^b^	1,333	83.21	162	10.11	107	6.68
Reduced PA^c^	928	57.93	157	9.80	517	32.27

*^a^EE, emotional exhaustion.*

*^b^CY, cynicism.*

*^c^PA, personal accomplishment.*

The prevalence of burnout was slightly higher in men than in women; however, this difference was not statistically significant (*P* = 0.0955). Chi-square tests indicated that there were significant differences in the prevalence of burnout among the medical staff in the different age groups (*P* = 0.0092). Respondents who were satisfied with patient-physician relationships and those who did not participate in the care of patients with COVID-19 showed lower rates of burnout (*P* < 0.0001 and *P* = 0.0001, respectively). More detailed information is showed in Table 1.

### Factors Influencing Burnout Among Medical Staff 1 Year After the COVID-19 Pandemic

Table 4 presents the results of the stepwise logistic regression analysis. Factors associated with burnout among the medical staff included “neutral” or “dissatisfied” patient-physician relationships (OR = 1.56, 95% CI: 1.18–2.06) and insufficient social support (OR = 5.47, 95% CI: 2.78–10.74 for low-level and OR = 2.51, 95% CI: 1.90–3.32 for medium-level). Mental resilience was negatively associated with burnout (compared with no burnout). Respondents who participated in the care of patients with COVID-19 had a higher risk of burnout symptoms than those who did not, and the OR was 1.44 (95% CI: 1.15–1.81).

**TABLE 4:** Multinomial logistic regression of factors associated with job burnout in medical staff.

Variables	OR^b^	95% CI^c^	*P*-value
**Patient-physician relationship (Ref^a^:** **Satisfied)**			
Neutral/Dissatisfied	1.56	1.18–2.06	0.0020
**Social support level (Ref. = High-level)**			
Low-level	5.47	2.78–10.74	<0.0001
Medium-level	2.51	1.90–3.32	<0.0001
**Mental resilience**	0.94	0.92–0.96	<0.0001
**Whether you directly participated in the care of patients with COVID-19 or not (Ref: No)**			
Yes	1.44	1.15–1.81	0.0015

*^a^Ref, reference.*

*^b^OR, odds ratio.*

*^c^CI, confidence interval.*

## Discussion

Overall, the results of our study suggest that burnout rates among the medical staff 1 year after the COVID-19 pandemic in Wuhan, China, remain uncertain. COVID-19 poses a strain on the entire healthcare system. Medical staff, particularly those in Wuhan, were under pressure to provide emergency aid for COVID-19 patients when available healthcare resources were limited in the early outbreak. The results showed that 37.39% of the medical staff experienced burnout, with mild burnout accounting for a large proportion. Additionally, reducing EE was the most common symptom among the three burnout dimensions. Previous studies have shown that many factors are associated with burnout among medical staff. These include work-related factors such as department, work hours, and work environment (relationships between colleagues and between doctors and patients) and sociological influences such as sex, age, and marital status (Rössler, 2012; Hu et al., 2021; Sampei et al., 2022). In our study, not directly participating in the care of COVID-19 patients and low social support were risk factors for burnout. In addition, mental resilience is a protective factor against burnout. These results suggest that even though 1 year had passed since the COVID-19 outbreak at the time of the survey, the negative impact on burnout among medical personnel remains unacceptable to nurses, and interventions targeting key populations are necessary.

Compared with studies at the beginning of the COVID-19 pandemic, the prevalence of burnout among medical staff appeared to have been mitigated 1 year after the start of the pandemic. Although the prevalence of burnout was still high in our study (37.39%), it was lower than the findings of a survey from more than 60 countries (37.39 vs. 51%) (Morgantini et al., 2020). Previous studies have suggested that anxiety/depression and burnout are related and perhaps overlap in symptoms (Koutsimani et al., 2019; Chen and Meier, 2021). Interestingly, a cross-national meta-analysis also showed that during the pandemic, China was the country with the lowest prevalence of anxiety and depression in the world (Castaldelli-Maia et al., 2021). Perhaps, it was this potential link between anxiety/depression and burnout that explains the lower prevalence of burnout in the current study sample than the incidence in the cross-national sample. In addition, this study showed that burnout prevalence among medical staff decreased 1 year after the COVID-19 outbreak compared to the beginning of the COVID-19 outbreak (Morgantini et al., 2020; Jang et al., 2021). To efficiently curb the spread of the disease, the Chinese government dispatched more than 42,000 medical staff to Wuhan to supplement the shortage of manpower. Compared with the beginning of the epidemic, the labor gap of local medical staff has been replenished with government support, thus easing the occupational stress of medical staff to some extent (Feng et al., 2020). Increased awareness and psychological preparedness for COVID-19 may have a positive effect on maintaining occupational safety and reducing occupational stress among medical staff (Liu et al., 2020; Huo et al., 2021).

The effects of COVID-19 on the three dimensions of job burnout were inconsistent. We found that the burnout symptoms of medical staff were mainly manifested as EE 1 year after the beginning of the COVID-19 pandemic, which was consistent with previous studies (Shanafelt et al., 2012; Hu et al., 2020). According to Maslach et al. (2001), the progression of the three dimensions of burnout varies over time; EE occurs first, leading to CY, followed by reduced PA. Currently, COVID-19 has undergone several generations of mutations, and the infectiousness and load of the virus have increased significantly (Krause et al., 2021; Salyer et al., 2021). Meanwhile, the worldwide epidemic trend of COVID-19 has not yet seen a turnaround. The number of infected patients increases explosively at the early stage (World Health Organization, 2020). Therefore, it is essential to clarify the long-term and dynamic effects of COVID-19 on the dimensions of burnout among medical staff. Although this was a cross-sectional study, it provides a reference for a longitudinal study.

Work factors, whether they were directly involved in the care of COVID-19 patients, doctor-patient relationships, and high social support in this study, were key influences on burnout. In our study, respondents who participated in the care of patients COVID-19 patients were 1.44 times more likely to experience burnout 1 year after the start of the COVID-19 pandemic than those who did not. This finding is consistent with a study conducted by Morgantini et al. (2020) in which the risk of infection among medical personnel was strongly associated with the risk of burnout. Previous studies have shown that medical staff who perceive insufficient social support are more susceptible to burnout (Zou et al., 2014, 2015). Similar results were observed in our study. This may suggest that in current stressful clinical settings, more emotional, informational, or companionship support should be provided to medical staff to reduce their burnout. In addition, our study found that 1 year after the COVID-19 outbreak, more than 80% of the Chinese medical staff reported that they were satisfied with patient-physician relationships, which is higher than the rates reported in previous studies (Wu et al., 2013, 2014). In addition, participants who were neutral/dissatisfied with the patient-physician relationship were 1.56 times more at risk of burnout than those who were satisfied. In Chen et al. (2013) study, unsatisfactory patient-physician relationships were strongly associated with high levels of burnout. Nonetheless, the mechanisms by which the patient-physician relationship affects symptoms of burnout in medical staff still require further studies.

In this study, mental resilience was negatively associated with burnout, which means that mental resilience mag be a protective factor against burnout in medical staff. Mental resilience is considered one of the mediators between traumatic events and psychological distress (Wagnild and Young, 1993). Studies, both before and during the early stages of an outbreak, have confirmed that mental resilience can reduce the negative effects of work stress (Arrogante, 2014; Serrão et al., 2021). Health-care workers with poor mental resilience have a relatively high risk of burnout (Hu et al., 2020). In future hospital management, relevant preventive and interventional psychological guidance to improve the mental resilience of medical staff should be an essential part of improving the psychological well-being of medical workers. In the context of a major public health event, such as COVID-19, how to carry out interventions for burnout in healthcare workers is a topic worth exploring. Building on the National Academy of Sciences’ recommendations for systematically improving clinician well-being, we encouraged monitoring burnout among medical staff over the long term. Second, the doctor-patient relationship and peer support should be improved to enhance the work environment. Finally, positive psychological interventions should be conducted to improve mental resilience against burnout among medical staff (National Academies of Sciences, 2019).

The main strengths of our study are as follows: First, our study investigated burnout status among medical staff 1 year after the beginning of the COVID-19 pandemic in Wuhan, the city most affected by the virus in China. Second, we determined the prevalence and possible factors influencing burnout among medical staff. This lays the foundation for future prospective studies assessing the level of burnout among medical staff.

Our study has several limitations. First, since this was a cross-sectional study, causal relationships between the presence of burnout and the variables could not be determined. Second, a convenient sampling technique was used to select only three hospitals in Wuhan, China; thus, the representativeness of the samples has certain limitations. In addition, the proportion of female nurses is relatively large; therefore, future studies should focus on the proportion of medical staff.

## Conclusion

Nearly two-fifths of the medical staff experienced burnout 1 year after the COVID-19 pandemic, with mild burnout accounting for a large proportion. Medical staff who perceive limited social support, respondents who report poor patient-physician relationships, and those who participate in the care of patients with COVID-19 should be the key intervention population. During and after the COVID-19 pandemic, timely and appropriate psychological interventions are urgently needed for the medical staff. In addition to monitoring the burnout status of key populations, it is essential to strengthening the assessment of the mental health of the entire healthcare team. In addition, it is important to play a good role in peer support when intervening and creating a good work environment. Through systematic interventions, burnout can be addressed by improving the mental resilience of the medical staff.

## Data Availability Statement

The original contributions presented in this study are included in the article/supplementary material, further inquiries can be directed to the corresponding author.

## Ethics Statement

The studies involving human participants were reviewed and approved by Research Ethics Committee in Tongji Medical College, Huazhong University of Science and Technology, Wuhan, China. The patients/participants provided their written informed consent to participate in this study.

## Author Contributions

WF and LZ: conceptualization and supervision. YL, FP, KZ, JM, and PZ: methodology. JZ and XB: formal analysis. YL, PZ, JZ, XB, and WF: investigation. WF: writing—original draft preparation. WF, YL, FP, KZ, JM, and LZ: writing—review and editing. All authors have read and agreed to the published version of the manuscript.

## Conflict of Interest

The authors declare that the research was conducted in the absence of any commercial or financial relationships that could be construed as a potential conflict of interest.

## Publisher’s Note

All claims expressed in this article are solely those of the authors and do not necessarily represent those of their affiliated organizations, or those of the publisher, the editors and the reviewers. Any product that may be evaluated in this article, or claim that may be made by its manufacturer, is not guaranteed or endorsed by the publisher.
